# Effects of Florfenicol on Intestinal Histology, Apoptosis and Gut Microbiota of Chinese Mitten Crab (*Eriocheir sinensis*)

**DOI:** 10.3390/ijms24054412

**Published:** 2023-02-23

**Authors:** Xinping Guo, Ziang Qian, Qiqi Pan, Yuqing Hu, Wangxin Mei, Xiumei Xing, Shaowu Yin, Jie Ji, Kai Zhang

**Affiliations:** 1 College of Marine Science and Engineering, Jiangsu Province Engineering Research Center for Aquatic Animals Breeding and Green Efficient Aquacultural Technology, Nanjing Normal University, Nanjing 210023, China; 2 Co-Innovation Center for Marine Bio-Industry Technology of Jiangsu Province, Lianyungang 222005, China

**Keywords:** florfenicol, gut barrier, apoptosis, gut microbiota, crab

## Abstract

Excessive use of antibiotics in aquaculture causes residues in aquatic animal products and harms human health. However, knowledge of florfenicol (FF) toxicology on gut health and microbiota and their resulting relationships in economic freshwater crustaceans is scarce. Here, we first investigated the influence of FF on the intestinal health of Chinese mitten crabs, and then explored the role of bacterial community in FF-induced intestinal antioxidation system and intestinal homeostasis dysbiosis. A total of 120 male crabs (48.5 ± 4.5 g) were experimentally treated in four different concentrations of FF (0, 0.5, 5 and 50 μg/L) for 14 days. Responses of antioxidant defenses and changes of gut microbiota were assessed in the intestine. Results revealed that FF exposure induced significant histological morphology variation. FF exposure also enhanced immune and apoptosis characteristics in the intestine after 7 days. Moreover, antioxidant enzyme catalase activities showed a similar pattern. The intestinal microbiota community was analyzed based on full-length 16S rRNA sequencing. Only the high concentration group showed a marked decrease in microbial diversity and change in its composition after 14 days of exposure. Relative abundance of beneficial genera increased on day 14. These findings illustrate that exposure to FF could cause intestinal dysfunction and gut microbiota dysbiosis in Chinese mitten crabs, which provides new insights into the relationship between gut health and gut microbiota in invertebrates following exposure to persistent antibiotics pollutants.

## 1. Introduction

As a major aquacultural country, commercial aquatic farming in China is predominantly based on intensive cultivation, which increases risks of disease outbreak and antibiotics abuse. According to a WHO report, China consumed nearly 50% of world’s antibiotics in 2013, approximately 162,000 tons, half of which came from animal farming [1]. Considering the durability and poor absorption after medication, overdoses of antibiotics have been confirmed to cause body deformity in aquatic animals, leave residues in food, and influence the emergence of antibiotic resistance genes for human pathogens [2].

Among antibiotics, as a third generation product of chloramphenicol, florfenicol (FF) is widely used in humans, veterinary clinics and aquaculture for its broad-spectrum benefits and lack of side effects [3]. This class of drugs blocks the process of bacteria protein translation by binding with ribosome 50S subunit to inhibit bacterial activity [4]. FF was first created as an alternative to chloramphenicol and thiamphenicol and was added in feed supplement to improve growth rate in aquaculture [5]. In North America, FF was applied in the therapy of enteric septicaemia of channel catfish (*Ictalurus punetaus*) [6]. However, abuse of FF caused environmental contamination. FF cannot be absorbed and metabolized completely in the gut, leading the antibiotic to be excreted from aquatic animals into water and sediment. There were also changes to the metabolisms of environmental microorganisms and the nitrogen cycle [7]. As an important source of animal protein, economic aquaculture organisms are mainly consumed by humans, which leads to an increase in FF residues and poses significant risks to human health. 

Previous toxicological studies of FF were mostly carried out in acute exposure. However, although FF was hydrolyzed and excreted quickly from organisms, many aquatic species continuously suffer through long periods of exposure to FF in aquatic environments, or even throughout their entire life cycle. Evidence demonstrated that chronic exposure to FF in high concentrations will result irreversible inhibit the hematopoietic system [8]. In addition, 21 days of FF treatment in adult zebrafish (*Danio rerio*) resulted in a substantial increase in glycolipid-related genes, leading to hepatic metabolic disorder [9]. Limited information has been found on the potential mechanism of FF toxicity in chronic environments and extreme concentrations, especially in invertebrates. Therefore, it is necessary to examine the chronic influence of FF residues on aquatic crustaceans more carefully.

In aquatic animals, as the major organ for immune defense and nutrient absorption, the intestine has direct contact with contacts and forms the first barrier of defense against the external environment. In addition, the intestine was also proven to be a major toxicity and metabolic target organ for antibiotic exposure in teleost [10]. He et al. [11] suggested that antibiotics exposure could disorganize epithelial structure, increase intestinal permeability, and then induce oxidative stress. In spite of this, previous studies have mainly focused on risk evaluation and exploring the potential functions of the liver and kidney when treated with antibiotics [12,13]. Recent research illustrated that intestine homeostasis is essential to organism health. Chronic exposure of sulfamethoxazole caused irreversible oxidative-stress-cascaded damage to gut and led to apoptosis in grass carp (*Ctenopharyngodon idella*) [14]. Moreover, the gut is known as a reservoir of antibiotic resistant genes under selective pressure from antibiotic exposure [15]. Collectively, these studies revealed that antibiotics can further damage intestinal health by destroying intestinal histological structure, elevating oxidative stress levels and inducing apoptosis. 

More evidence demonstrated that gut microbiota has a profound relationship with host immunomodulation, physiology and pathogen defense [16]. Commensal gut microbiota regulate host metabolism in multiple ways, including synthesizing and absorbing nutrients, strengthening gut barrier integrity, producing beneficial metabolites and other functions [17]. However, physical and chemical environmental factors (e.g., oxygen, heavy metals and pesticides) can affect bacterial community diversity and richness, impairing the intestinal barrier and triggering a series of metabolic diseases [18,19,20]. Recent studies suggest that antibiotic exposure also adversely affects the intestinal function of aquatic organisms. Qian et al. reported that three veterinary antibiotics (doxycycline, oxytetracycline and FF) which were received widespread use in China caused gut microbiota dysbiosis and dysfunction in zebrafish (*D. rerio*), leading to metabolic disorders [9]. Additionally, sulfamethazine changed the composition of gut microbial communities, downregulated alpha diversity and induced oxidative stress in marine medaka (*Oryzias melastigma*) during 30 days of oral exposure [21]. Thus, a functional and stable gut microbiota is critical for host physiology and intestinal homeostasis. These studies were also based mainly on short-read amplicons, producing higher proportion of inaccurate classification, especially at genus and species level. Nowadays, newly developed full-length 16S rRNA gene sequencing methods could cover V1-V9 hypervariable regions and conduct a highly resolved classification of intestinal community composition [22]. 

Crustaceans are vulnerable to antibiotics. Accumulated FF in crustaceans can cause hepatopancreatic structural damage and oxidative stress [23], immunosuppressive effect [24] and biomolecule damage [25]. The Chinese mitten crab (*Eriocheir sinensis*) is an economical freshwater aquatic animal widely bred in China with an annual production of more than 775,887 tons in 2021, according to the *China Fishery Statistical Yearbook*. The crab suffers from various antibiotics via intensive farming patterns, diet and the deterioration of the ecological environment. More importantly, *E. sinensis* is also reported to be a model organism for monitoring the ecotoxicological effects of antibiotics in aquatic environment [26]. However, to our knowledge, systematical studies of FF-induced toxicity and gut barrier imbalance in the Chinese mitten crab are scarce. 

Thus, considering the crucial role of gut microbiota in host intestinal homeostasis, the aim of this study was to identify chronic toxic effects of FF on the intestinal health of *E. sinensis* under environmental and extreme concentrations. Chinese mitten crabs were exposed to 0.5, 5 and 50 μg/L FF for a 14-day period. Toxicological changes in gut histological morphology, oxidative stress and apoptosis characteristics were evaluated. In addition, full-length 16S rRNA sequencing technology was carried out to analyze microbiota composition alterations in sides of exposure time and concentration. Results of this study will facilitate a better understanding of FF-induced toxicology on gut health of crustaceans.

## 2. Results

### 2.1. Intestinal Histomorphology Alterations after FF Exposure 

**Figure 1:**
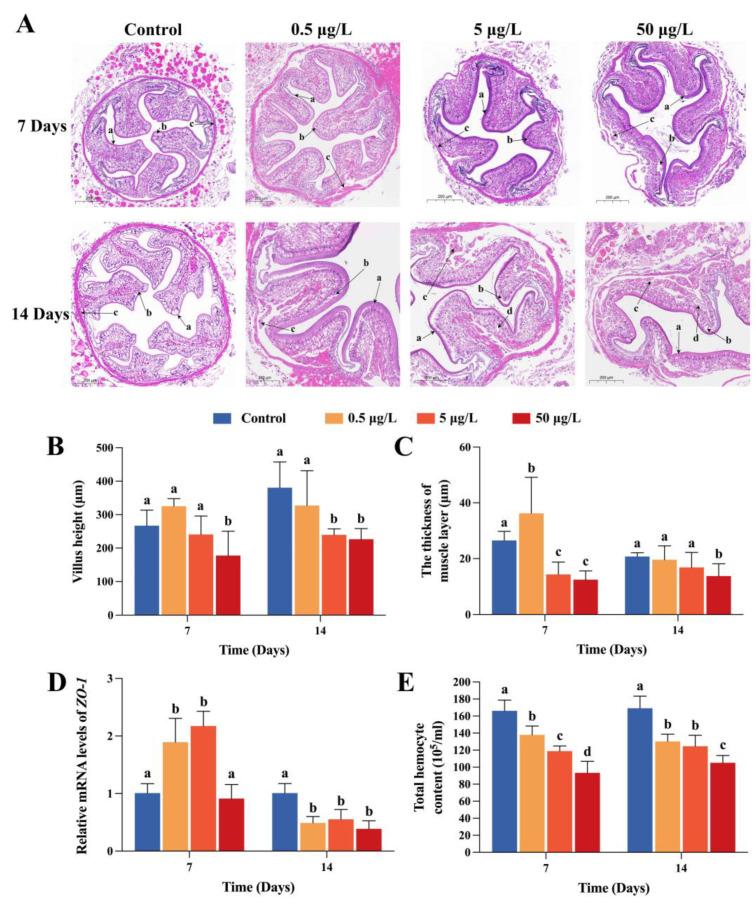
FF-induced intestinal damages in Chinese mitten crab. (**A**). Intestinal histological variation taken by hematoxylin-eosin assay. Magnification is 200× and the scale bar is 200 μm. Letters in the figure represent (a) intestinal peritrophic membrane; (b) epithelial cells; and (c) muscle layer; (d) vacuole. (**B**) Villus height. (**C**) The thickness of muscle layer. (**D**) mRNA levels of *ZO-1*. (**E**) Total hemocyte content. Data was analyzed based on six separate samples and presented as the mean ± SEM. Here and hereafter, different letters (a, b, c, d) indicate significant difference among control and three FF exposed groups (*p* < 0.05).

Intestinal morphological changes were monitored in crabs with H&E staining between control and FF treatment groups. Clearly damaged characteristics were observed. As shown in Figure 1A, the intestine in the control group had normal morphology, well arranged epithelial cells and peritrophic membrane. However, crabs treated with 50 μg/L FF exhibited markedly reduced villus lengths and significantly thinner muscle layers (*p* < 0.05) at either day 7 or 14 (Figure 1B,C). Although the villus length shortened slightly, the apical epithelium of intestinal villi was shed in LC and MC groups. Furthermore, the number of vacuole increased as the FF concentration increased at day 14. Transcriptional changes of *ZO-1* were detected to indicate the changes of gut permeability. As apparent from Figure 1D, the transcripts of *ZO-1* were all augmented in LC and MC groups on day 7 and strongly inhibited on day 14 in the three treated groups (*p* < 0.01). 

### 2.2. Effects of FF Exposure on THC and FF Contents in Intestine

**Figure A1:**
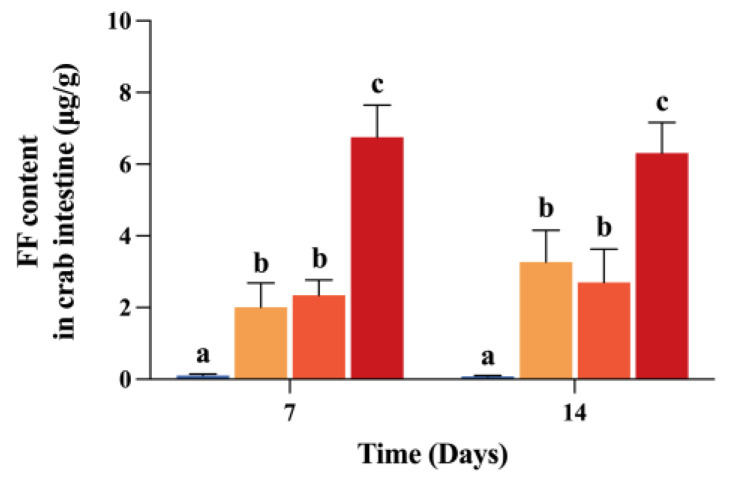
FF content in intestine. Different letters (a, b, c) indicate significant difference among control and three FF exposed groups (*p* < 0.05).

Compared with the control group, the THC noticeably decreased even in the LC group at day 7 and 14 (Figure 1E). In addition, the amount of THC was inversely proportional to the exposure concentration of FF. After detection of FF by HPLC, the concentration of FF showed a dose-dependent increase in the model of the crab intestine (Figure A1). In details, the FF contents in HC group were dramatically higher than those in LC and MC groups (*p* < 0.05). No significant changes were observed between LC and MC group. 

### 2.3. Effects of FF Exposure on Apoptosis

**Figure 2:**
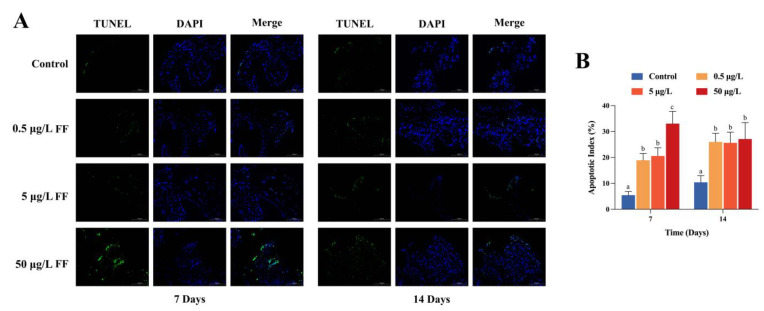
Effects of FF-exposure on apoptosis characteristics in Chinese mitten crab intestine. (**A**) Representative images in four groups. Nuclei of cell (blue) were depicted by DAPI staining, and potentially apoptotic cells (green) were indicated by TUNEL staining. Magnification is 200× and the scale bar is 100 μm. (**B**) Apoptosis index. Microscope fields were used to count total numbers of nuclei and TUNEL-positive cells, and the ratio was calculated. All data are expressed as mean ± SEM (n = 6). The different lowercase letters above each bar represent significant difference (*p* < 0.05).

TUNEL assay was used to evaluate the effect of FF exposure on apoptosis (Figure 2A). Obvious apoptosis characteristics were observed in FF treated groups at day 7 and 14, although weak signals were detected in the control group. The apoptosis index was dramatically increased in experimental groups (peak at 33.05 ± 4.71%) compared with the control group (5.46 ± 1.41% and 10.42 ± 2.57%) on both days 7 and 14 (*p* < 0.05) (Figure 2B). In day 7, the apoptosis index upregulated in a dose-dependent pattern, with the HC group showing the highest rate. Apoptotic cells first appeared in epithelial cells and were then located around the lamina propria. 

**Figure 3:**
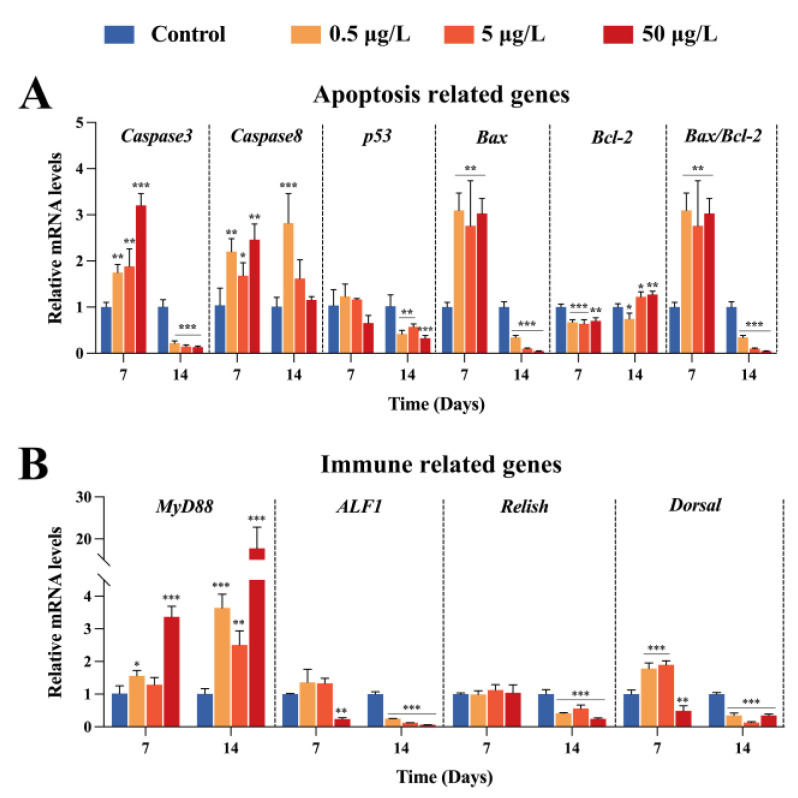
mRNA expression levels of apoptosis (**A**) and immune (**B**)-related genes in intestine. The mRNA amounts of detected genes were normalized to the values of corresponding internal gene. The asterisks “*” indicate statistical significance compared to the control group (* *p* < 0.05, ** *p* < 0.01 and *** *p* < 0.001). Data are shown as mean ± SEM (n = 3).

Relative expression of apoptosis-related genes in gut was tested to evaluate the influence of FF exposure on cell death (Figure 3A). At 7 days after exposure, the mRNA levels of *Caspase3* and *Caspase 8* were significantly elevated as the FF levels increased compared to the control (*p* < 0.05). Similar results were obtained in *Bax*. No significant changes exist in *p53* gene. In contrast, as an antiapoptotic-related gene, the transcripts of *Bcl-2* noticeably decreased (*p* < 0.01) in the three FF treated groups, indirectly leading to the upregulated ratio of *Bax*/*Bcl-2* (*p* < 0.01). 

However, on day 14, the relative expression pattern of *Caspase3*, *Bax* and *Bcl-2* showed opposite trends. Among them, even in the LC group, the mRNA levels of *Caspase3* and *Bax* were dramatically decreased (*p* < 0.001). Similarly, the ratio of *Bax*/*Bcl-2* were of 2.9-fold, 9.7-fold and 21.3-fold lower than the control group in the LC, MC and HC groups, respectively. Simultaneously, the relative expression of *p53* was strongly inhibited by FF with increased dosage (*p* < 0.01 or *p* < 0.001). In the case of *Caspase8*, significant change only occurred in the LC group compared to control group (*p* < 0.001), which was different than the profile on day 7. 

### 2.4. Effects of FF Exposure on Expression of Genes Related to Immune Response

Levels of key genes in immune response were determined via qRT-PCR analysis, which is displayed in Figure 3B. The expression levels of *MyD88* were greatly upregulated in three treated groups and peaked in the HC group (*p* < 0.001), either at day 7 or day 14 postexposure. Regarding the transcription of antimicrobial peptides, *ALF1*, *Relish* and *Dorsal* mRNA downregulated sharply (*p* < 0.001) on day 14, showing a high degree of consistency. These results revealed the influence of FF on Chinese mitten crab immunity. 

### 2.5. Intestinal Oxidative Stress Responses 

**Figure 4:**
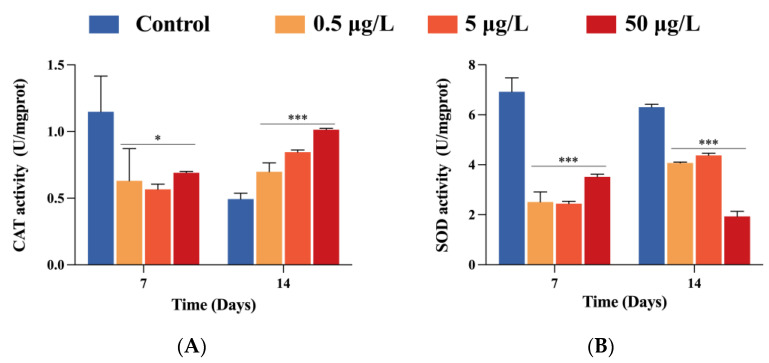
Effects of FF-exposure on the activity of CAT (**A**) and SOD (**B**). Bars represent the mean ± SEM (n = 5). The asterisks “*” indicate statistical significance compared to the control group (* *p* < 0.05 and *** *p* < 0.001).

To access the oxidative stress levels induced by FF exposure, antioxidant enzymes CAT and SOD activities were measured. Compared with the control group, crabs in three FF treatment groups showed lower CAT activities on day 7 (*p* < 0.05) (Figure 4A). In contrast, CAT contents increased significantly (*p* < 0.001) in dose-dependent manner on day 14. Moreover, SOD activities stayed suppressed (*p* < 0.001) from 7 to 14 days compared to group control (Figure 4B).

### 2.6. Intestinal Microbiota Changes after 14 Days of FF Exposure with Different Concentrations

#### 2.6.1. Richness and Diversity

**Figure A2:**
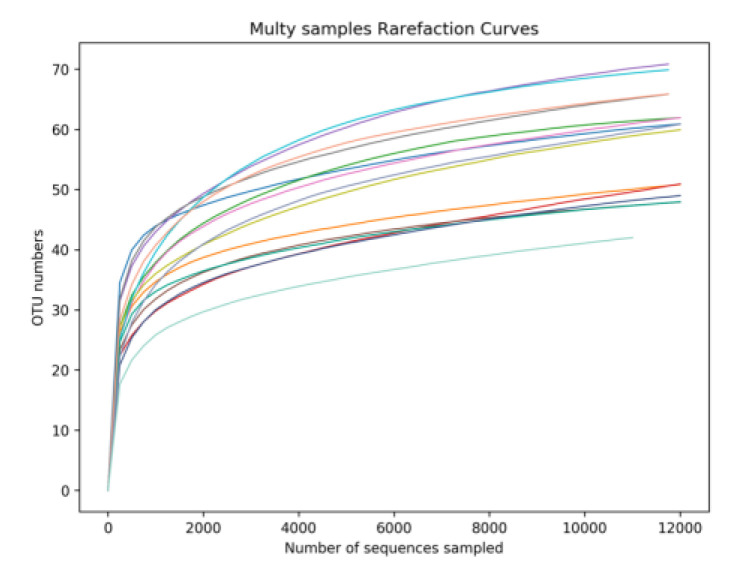
Rarefaction curves for all gut bacteria community. Each curve in the graph represents different crabs. As the sequencing depth increased, the number of gut bacteria increased the relative abundance increased in rarefaction curves.

Full-length 16S rRNA gene high-throughput amplification was employed in 12 intestinal content samples via the PacBio platform. A total of 194,629 original CCS sequences (range from 12,873 to 13,096) were produced in the current microbiome analysis. The Good’s coverage of all samples exceeded 99.91%, suggesting that the sequencing production could represent the majority of bacteria in crab intestine of this study. The rarefaction curve (Figure A2) also reflected sufficient sequencing depth.

**Figure 5:**
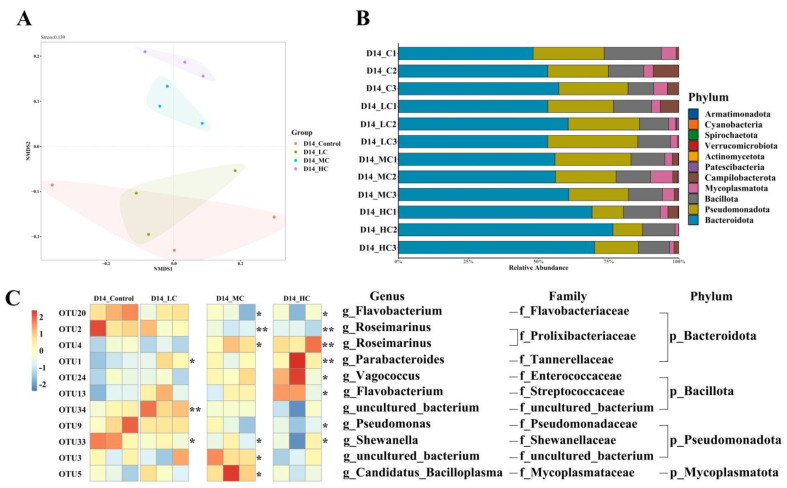
High concentration of FF induced significant changes in microbiota richness on day 14. (**A**) Relative abundance of bacterial at phylum level (n = 3). (**B**) NMDS based on Bray–Curtis distance matrix of OTUs. (**C**) Heatmap analysis of 11 OTUs. The color bar of each OUT in each sample is shown. The taxonomy of OTUs (genus, family and phylum) is depicted on the right. Differences were detected by one-way ANOVA. D14 denotes the samples exposed on day 14. C, LC, MC and HC denote control group, low concentration group (0.5 μg/L), median concentration group (5 μg/L), and high concentration group (50 μg/L), respectively. The asterisks “*” indicate statistical significance compared to the control group (* *p* < 0.05 and ** *p* < 0.01).

Alpha diversity of the intestinal microbes in control, LC, MC and HC group after 14 days of FF exposure were shown in Table 1. Compared with those in control group, Shannon and Simpson indexes were remarkably downregulated only in the HC group (*p* < 0.05), while the Chao1 and ACE indexes were statistically the same in all four groups, indicating no changes in bacterial richness. An NMDS plot based on Bray–Curtis dissimilarity was generated to visualize the β-diversity among groups (Figure 5A). Interestingly, compared with MC and HC group, LC group had a tendency toward variation distribution in community composition approach at the control level. 

#### 2.6.2. Intestinal Microbial Composition

The microbiota compositions at the phylum level were further analyzed. As shown in Figure 5B, *Spirochaetota* was newly detected in the gut microbial communities of MC and HC groups, although the relative abundance was low with the value of 0.02%. The top five most abundant phyla were *Bacteroidota* (52.82%), *Pseudomonadota* (23.86%), *Bacillota* (23.86%), *Mycoplasmatota* (4.53%) and *Campilobacterota* (4.73%) in the control group. Metastats analysis suggested that changes of bacterial phyla only occurred in the HC group when the *Bacteroidota* sharply increased to 71.82% (*p* < 0.001) and *Pseudomonadota* significantly decreased to 12.44% (*p* < 0.01) compared to the control. No significant difference was detected in the LC and MC groups. 

Eleven OTUs which showed significant differences among the four groups were chosen for heatmap analyses (Figure 5C). Compared to those in the control group, FF exposure decreased the proportions of *Flavobacterium*, *Roseimarinus* (out2), *Pseudomonas* and *Shewanella* (*p* < 0.05). Seven OTUs increased significantly in the antibiotic exposed groups, and those OTUs were distributed in *Bacteroidota*, *Bacillota*, *Pseudomonadota* and *Mycoplasmatota*. Interestingly, OTU2 and OTU4, which both belong to *Roseimarinus*, had opposite directions of variation. We also noticed that most significant alterations occurred in the HC group, including OTU20 (*Flavobacterium*), OTU2 (*Roseimarinus*), OTU4 (*Roseimarinus*), OTU1 (*Parabacteroides*), OTU24 (*Vagococcus*), OTU13 (*Lactovum*), OTU9 (*Pseudomonas*) and OTU33 (*Shewanella*). These results indicated that high concentration exposure to FF influenced the intestinal microbial composition more deeply than other two concentrations as compared to the control on day 14. 

### 2.7. Comparison of Intestinal Microbiota Changes between Day 7 and Day 14 after Low Concentration FF Exposure

#### 2.7.1. Biodiversity Analysis

**Figure 6:**
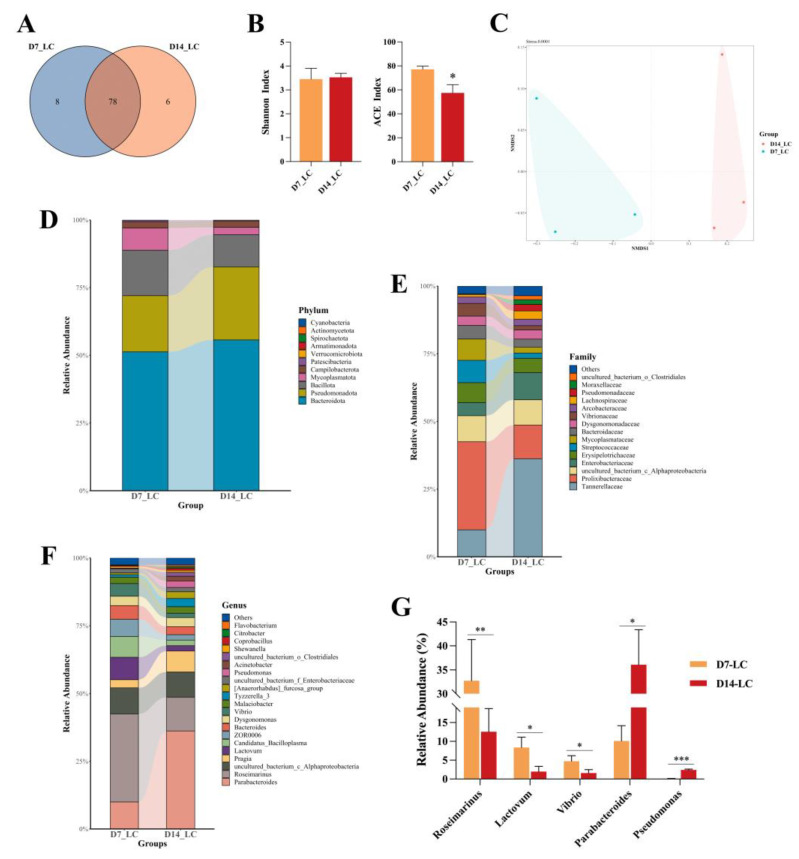
Low concentration of FF-exposure altered the diversity of the intestinal microbial community in crabs between day 7 and day 14. (**A**) The Venn diagram analysis of OTUs. (**B**) Alpha diversity: Shannon index and ACE index. (**C**) NMDS based on Bray–Curtis distance. (**D**–**F**) Relative abundance of gut bacterial composition at phylum, family and genus level. (**G**) Metastats analysis of different phyla in intestinal microbiota at the genus level. The asterisks “*” indicate statistical significance compared to the control group (* *p* < 0.05, ** *p* < 0.01 and *** *p* < 0.001). Bars represent the mean ± SEM (n = 3).

To compare the alterations of the intestinal microbial community after 0.5 μg/L of FF exposure at 7 and 14 days, full-length 16S rRNA sequencing was conducted. The Venn diagram (Figure 6A) reflects that 78 OTUs were co-owned between day 7 and day 14, and the unique OUTs were eight and six, respectively. Community diversity was determined by Shannon indices and showed no significant difference between two groups (*p* > 0.05, Figure 6B). However, the bacterial richness markedly downregulated with the prolonging of FF exposure (*p* < 0.05). The nMDS analysis further confirmed that differential microbiota patterns existed in crabs from those two groups (Figure 6C). 

#### 2.7.2. Microbial Community Composition

The taxa of dominant bacteria were analyzed at the phylum, family and genus levels. At the phylum level (Figure 6D), the dominant phyla in the two groups were *Bacteroidota*, *Pseudomonadota*, *Bacillota*, *Mycoplasmatota* and *Campilobacterota*. The abundance of these five detectable phyla did not significantly alter. Notably, *Spirochaetota* was absent in the 14-day treatment. Moreover, at the family level, *Tannerellaceae*, *Lachnospiraceae*, *uncultured_bacterium_o_Clostridiales* and *Pseudomonadaceae* were strongly enriched (*p* < 0.05). In addition, long-term FF exposure noticeably reduced the abundance of *Streptococcaceae*, *Vibrionaceae* and *Prolixibacteraceae* (*p* < 0.05, Figure 6E). At the genus level, 68 genera were identified from all samples, and the top 21 relative abundances are shown in Figure 6F. Metastats analysis showed that *Roseimarinus*, *Lactovum* and *Vibrio* were all decreased significantly on day 14 (*p* < 0.05 or *p* < 0.01), while *Parabacteroides* and *Pseudomonas* were more abundant on day 14 compared to day 7 (*p* < 0.05 or *p* < 0.001, Figure 6G). 

#### 2.7.3. Linear Discriminant Analysis

**Figure 7:**
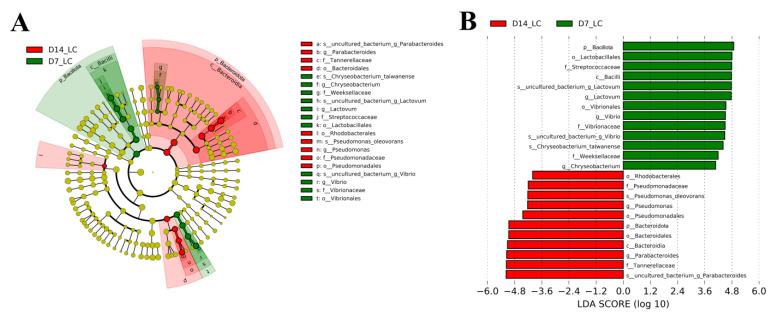
Intergroup variation of the intestinal microbes of *E. sinensis* in low concentration FF exposure. (**A**) Linear discriminant analysis effect size (LEfSe) of microbial abundance in LC group. (**B**) LDA score of LEfSe-PICRUSt from phylum to species level. Only the taxa with a linear discriminant analysis threshold score of 4.0 are shown.

LEfSe was employed to indicate the high-dimensional biomarkers in the LC group that was exposed for 7 and 14 days. As described in the cladogram (Figure 7A), two bacterial taxa contributed to the 7-day group, including *Bacillota* and *Vibrionales*. LEfSe analysis also revealed that the relative abundance of *Bacteroidota* and *Pseudomonadales* were more abundant in long-term FF exposure. At the genus level, LDA analysis with cut-off value of 4.0 identified *Lactovum*, *Vibrio* and *Chryseobacterium* as the indicator bacteria in the intestine of crabs in 7-day exposure, whereas *Pseudomonas* and *Parabacteroides* were the signature bacteria in the 14-day group (Figure 7B). 

#### 2.7.4. Microbial Function Prediction

**Figure 8:**
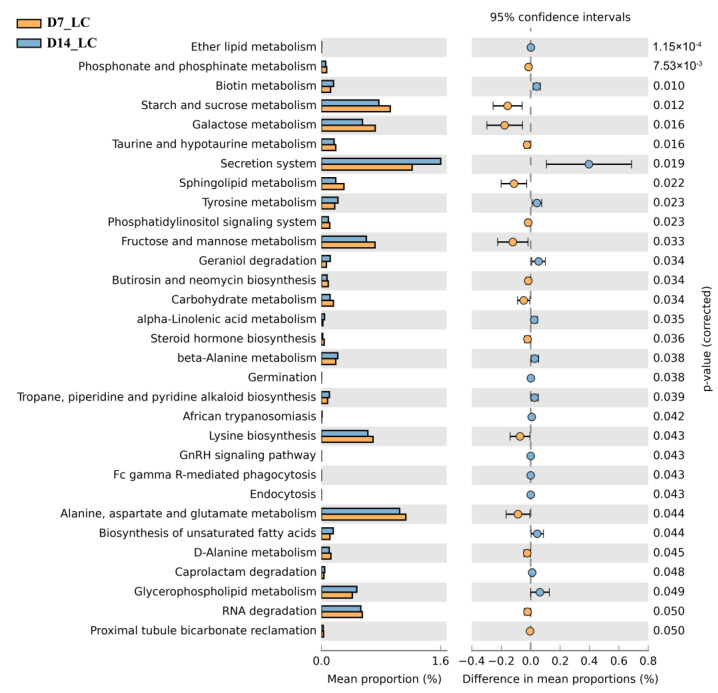
KEGG pathway analysis of intestinal microbial metabolism in crabs after low concentration FF exposure.

To further understand the changes in metabolic functions, PICRUSt was performed in LC group (Figure 8). Observation of metabolic pathway revealed that “secretion system” was the dominant microbial function detected in LC groups during exposure. Interestingly, the proportion of “glycerophospholipid metabolism” and “biosynthesis of unsaturated fatty acids” expanded with the prolonging of FF treatment, suggesting that FF could affect host lipid metabolism. Additionally, pathways in glycose metabolism, including “starch and sucrose metabolism”, “galactose metabolism”, and “fructose and mannose metabolism”, were enriched in the early exposure stage. 

### 2.8. Correlation between Gut Microbiota and Chinese Mitten Crab Gut Health

**Figure 9:**
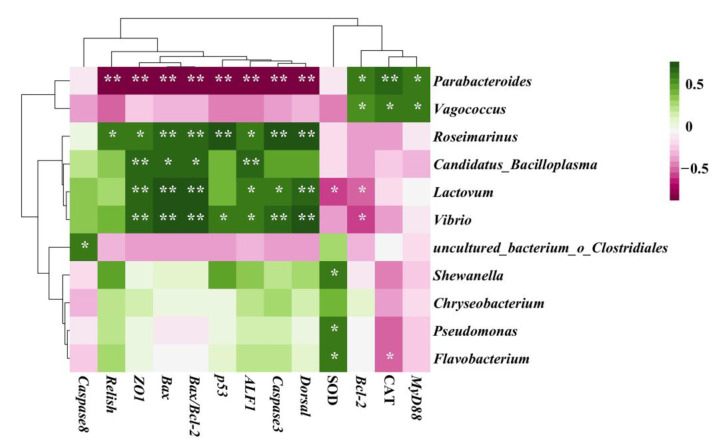
Correlations between intestinal genera and crab gut health. The asterisks “*” indicate statistical significance compared to the control group (* *p* < 0.05 and ** *p* < 0.01).

After combining the results of the metastats analysis and the genus classification level in LEfSe analysis, Spearman correlations between gut microbiota and biochemical indices of the Chinese mitten crab were conducted. As described in Figure 9, apoptosis-related genes and antimicrobial peptides were positively correlated with *Lactovum*, *Vibrio* and *Roseimarinus*, and negatively associated with *Parabacteroides* (*p* < 0.05 or *p* < 0.01). In addition, *Parabacteroides* and *Vagococcus* showed strong positive correlations with CAT activities and expression of *MyD88* and *Bcl-2* (*p* < 0.05 or *p* < 0.01). 

## 3. Discussion

Antibiotics are widely used in aquaculture as feed additives and therapeutics to promote growth rate and prevent bacterial infections, respectively. Recently, consumption of antibiotics has raised concerns for its prevalence and high residue rate in foods, such as milk, eggs and aquatic products [27]. Previous studies reported that aquatic animals are more susceptible to low concentration of antibiotics, which induced antibiotic resistance [28]. Excessive antibiotics destroy intestinal structure in teleost, leading to gut dysfunction [10,29]. However, relationships between crustacean intestine and microbiota under environmental and extreme doses remains uncertain. Here, an florfenicol poisoning model was conducted in Chinese mitten crabs to explore the potential mechanism of florfenicol-induced intestinal damage. In the present study, histopathological structure, oxidative stress and apoptosis pathways, as well as gut microbial alteration, were examined in adult *E. sinensis* exposed to 0, 0.5, 5 and 50 μg/L FF.

As a major organ for nutrient digestion and absorption, the intestine of aquatic animals is also intimately involved in host metabolism, immune defense and other crucial activities. All of these procedures require a healthy intestinal structure and complete intestinal barrier [30]. Physical, biochemical and immunological barriers are the three main components of an intact intestinal barrier. According to histopathologic analysis, significant changes were observed in intestinal villus architecture with apical epithelial shedding and villi shortening, implying increased permeability and susceptibility of intestinal barrier after FF exposure. Exposure to 100 μg/L FF also resulted in intestinal morphology and structure damage to zebrafish [9]. Furthermore, expression levels of *ZO-1* were detected. Working as a tight junction protein, ZO-1 formed a physical barrier by tightening intestinal epithelial cells to strengthen intestinal function, which caused damage due to increased intestinal permeability [31]. As expected, FF inhibited *ZO-1* expression after 14 days of exposure. Previous studies found that adding 2.0 g/kg oxytetracycline to the diet of Nile tilapia (*Oreochromis niloticus*) reduced the expression of *ZO-1* and *CLDN3* [29]. Qian et al. reported that exposure to environmentally relevant concentrations of sulfamethoxazole (0.3 μg/L) suppressed *ZO-1* expression and induced inflammation and apoptosis in grass carp [9]. However, *ZO-1* mRNA levels significantly increased in LC and MC groups at 7 days, indicating an epithelial structure reinforcing phenomenon, which is generally consistent with the histopathologic analysis. Antibiotics were used early as feed additives to promote the growth trait in aquaculture, which might be involved in *ZO-1* upregulation on day 7. Additionally, in crustaceans, hemocytes are closely related to innate immune functions. Thus, the immunological status of aquatic animals could be directly reflected through THC. In the present study, FF-treated crabs showed a significant drop in THC. Similar results were reported in mud crabs (*Scylla paramamosain*) [32]. Decreased HTC might be caused by cell apoptosis [33]. These results suggest that high FF exposure increases intestinal permeability and damages the intestinal barrier, leading to serious destructions in crabs. The extent of intestinal damage is limited in environmental dose exposure. 

Antibiotics-induced excessive reactive oxygen species (ROS) can elevate oxidative stress in aquatic animals, leading to severe oxidative stress damage [12,34]. As the first part of the antioxidant system, SOD and CAT can eliminate overproduction of ROS under normal conditions, and therefore reflect the overall antioxidant status of organisms. Previous studies on swimming crab (*Portunus trituberculatus*) reported noticeably decreased SOD activities after intravenous dosing treatment of FF [25]. Chen et al. [35] observed significant decrease of SOD and CAT activities in a dose-dependent manner after 6 days of sulfamethoxazole treatment in marine mussels (*Mytilus galloprovincialis*). Similarly, in the present study, activities of SOD and CAT were both significantly decreased after 7 days of exposure in three FF exposed groups, suggesting that even the ng/L concentration would cause too much ROS to damage the antioxidant system. Nevertheless, as the FF stress was prolonged, different profiles appeared between SOD and CAT, with a dramatic increase of CAT activity on day 14. CAT has the ability to degrade hydrogen peroxide into molecular water and oxygen. The recovery of CAT activity revealed that oxidative stress of organism is weakened. In general, the reduction of CAT and SOD suggested the degradation of antioxidative status in Chinese mitten crab under FF exposure. Thus, these antioxidative enzymes are widely used as biomarkers in ecological risk assessment [36]. In present results, CAT showed a dose-dependent manner which indicated the feasibility and high efficiency as biomarker for florfenicol stress in crustaceans. 

In the TLR signaling pathway, MyD88 activates a string of downstream signaling cascades, and finally increases the expression of NF-κB [37]. NF-κB is a key protein in host inflammation and innate immune response in invertebrates [38]. Evidence suggests that high doses of maduramicin contribute to intestinal inflammatory in crayfish (*Procambius clarkii*) [39]. In this study, *MyD88* mRNA transcripts were maintained at a relatively high level during all 14 days of exposure, suggesting the occurrence of intestinal inflammatory response in crabs. In addition, AMPs, the major antibacterial effectors activated by NF-kB pathways, would be secreted into extracellular space to resist environmental pollutions stress and microbial invaders in crustaceans [40]. In the present study, long-term exposure of FF markedly inhibited the transcription of three AMPs, namely, *ALF1*, *Relish* and *Dorsal*. In contrast, at 7 days of exposure, significant changes were found only in the HC group, which is consistent with the results of THC. 

To reflect the degree of injury in intestine tissue, cytometric TUNEL assay and expression of apoptosis-related genes were conducted. Previous investigation revealed that the apoptosis rate of zebrafish embryonic cells was enhanced with a dose-dependent pattern of 4-Epianhydrotetracycline [41]. The apoptosis index markedly upregulated at both day 7 and 14 compared with the control group, indicating that FF exposure not only damaged the oxidative system but also induced intestinal cell apoptosis. Correspondingly, this point was proved by the expression pattern of apoptotic-related genes. Zhao et al. reported that the Bax/Bcl-2 protein ratio was upregulated in grass carps after exposure to cypermethrin or/and sulfamethoxazole in a 42-day period [42]. Likewise, the ratio was almost threefold higher comparing to the blank control after 7 days of FF exposure. However, as the exposure was extended to 14 days, completely reversed expression profiles of these apoptosis-related genes were observed. Apoptosis functions in programmed cell death by eliminating excess, infected or damaged cells [43]. Combined with SOD activities, we speculate that this phenomenon might be correlated with the regulation of intestinal homeostasis.

Accumulating studies illustrated that gut microbiota has a close relationship in maintaining host intestinal barrier integrity, oxidate stress and immunity in aquatic animals when facing with environmental contaminants [9,20]. Here, we used full-length 16S rRNA sequencing technology to investigate dynamic changes on crab gut microbiota diversity at dosage and time dimension. After 14 days of exposure, α diversity index was only remarkedly decreased in the HC group compared with control, indicating that intestinal homeostasis worked in the LC and MC groups. At the phylum level, relative content of *Bacteroidota* increased obviously while *Proteobacteria* decreased after 50 μg/L FF exposure, which was in accord with one previous study on *E. sinensis* subjected to imidacloprid [20]. Together, compared with bacterial quantity, bacterial composition and diversity were more sensitive to high FF exposure. After 75 mg/kg per day oxytetracycline oral treatment, only bacterial composition altered in Atlantic salmon (*Salmo salar*) intestine [44]. This could be explained by increased abundance of antibiotic-resistant bacteria and drop in antibiotic-sensitive bacteria. 

OUTs in LC group at 7 and 14 days were relatively few compared to previous studies of antibiotic exposure [9]. This might be attributable to FF residues which eradicated susceptible microorganisms [44]. Furthermore, in contrast with alpha diversity in the four groups at day 14, bacterial quantity indicated by ACE index was changed dramatically. Although no statistical significance changes were seen at the phylum level, the trend of bacterial composition was highly consistent with the results of Hong et al. [20], indicating a conserved biological function of gut microbiota. *Bacteroidota* participate in immunomodulation and lipid metabolism [45]. Likewise, microbial function also predicted that lipid metabolism is the dominant function of gut bacteria in long-term exposure. Absence of *Bacteroidota* and induced *Bacillota* were proved to be associated with a deteriorating of non-alcoholic fatty liver disease leading to hepatic steatosis [46]. The *Bacillota*/*Bacteroidota* ratio, correlated with metabolic disorders, was higher in day 14. At the family level, the relative abundance of *Tannerellaceae* increased with prolonged exposure. The presence of the *Tannerellaceae* family is reported to have beneficial effects in therapy of collagen-induced mouse arthritis [47]. In addition, some other nondominant families in gut microbiota also changed in FF treated crabs. *Pseudomonadaceae*, regarded as a degrader of organic pollutants [48], was significantly increased at day 14, implying the removal of antibiotics from crabs. Ratio of *Lachnospiraceae*/*Streptococcaceae* is known to be negatively associated with metabolic disorder and proinflammatory processes [49]. A similarly increased ratio was observed in our findings. Results from metabolic function prediction demonstrated that this change is responsible for glycolysis and lipid metabolism in crab intestines, giving us a hint about the function of gut microbiota in FF metabolism. 

Finally, we explored the bacterial composition at the genus level. *Parabacteroides*, regarded as beneficial bacteria in amino acid metabolism that also participate in proteolytic functions [50], showed strong augmentation in crabs with long-term exposure in the present study. Notably, the abundance of opportunistic pathogens *Vibrio* was significantly downregulated. This might be attributed to the broad-spectrum antibacterial function of FF. Our results are in accordance with a report from [51], which said that nanoplastics could invert the proportion of *Parabacteroides* and *Vibrio* in the large yellow croaker (*Larimichthys crocea*). Moreover, *Pseudomonas* can efficiently decompose organic contaminants [52]. Thus, based on the function of metabolizing xenobiotic compounds, we speculate that gut microbiota contributes to host defense by degrading and detoxifying FF in aquatic crustaceans. Correlation analysis provides the associations between physiological indicators and gut bacteria during FF exposure. In this study, *Lactovum*, *Vibrio* and *Roseimarinus* were positively associated with *ZO-1*, AMPs and apoptosis-related genes, while *Parabacteroides* showed strongly negative correlation. However, little is known about *Lactovum* and *Roseimarinus* in aquatic animals. These two genera may have similar functions with *Vibrio* through correlation analysis. Underlying mechanisms of host-gut microbiota interactions need to be further confirmed by in vitro experiments.

## 4. Materials and Methods

### 4.1. Chemicals

Florfenicol (FF, CAS No.: 73231-34-2, 98% in purity, Aladdin, China) was purchased from Aladdin Co., Ltd., Shanghai, China. FF is dissolved in dimethyl sulfoxide (DMSO, Solarbio, Beijing, China) with a concentration at 1 mg/mL as a stock solution. At days 7 and 14 after exposure, FF contents in the intestines were detected by HPLC. 

### 4.2. Crabs, Florfenicol Exposure and Sample Collection

Healthy crabs (48.5 ± 4.5 g) were collected from a local farm in Gaochun, Jiangsu Province, China. To eliminate the influence of sex differences, only male individuals were used in the experiment. All experimental animal procedures were in accordance with the principles of Institutional Animal and Use Committee of the Nanjing Normal University.

In total, 120 crabs were randomly divided into four triplicate groups and distributed in 12 tanks (60 × 40 × 30 cm; length, width, height) that each contained 10 L water. Before exposure, all crabs were cultured for two weeks to acclimatization to the following conditions: 12 h light/dark cycle; pH: 7.9 ± 0.3; temperature: 24.5 ± 2.0 °C; dissolved oxygen: 6.3 ± 0.7 mg/L; ammonia nitrogen < 0.2 mg/L. Crabs were fed with commercial feed (Tongwei, China) twice a day at the ration of 2% of body weight. 

According to the environmental concentrations in surface water and sediment of aquaculture area [53] and previous studies [9], concentrations of FF conducted in the present immersion exposure experiment were set to 0.5, 5 and 50 μg/L, referred to as low concentration (LC), median concentration (MC) and high concentration (HC), respectively. The group without any FF addition was used as control, identified as C in the following text. During the treatment, one third of the water was replaced daily, and FF was added in the treated tanks to maintain the concentration at the initial levels throughout the experiment. After 7 and 14 days of consecutive exposure, five crabs from each group were captured and the intestine of each individual was quickly sampled for enzyme activity determination and RNA isolation. Simultaneously, the contents of intestine were also squeezed for microbiome analysis. Samples were frozen in liquid nitrogen immediately and then stored at −80 °C for further analysis. 

### 4.3. Histopathological Analysis

Intestines from two crabs were randomly selected from each group at days 7 and 14 postexposure, and then fixed in paraformaldehyde (4%) solution for 24 h. Solid wax blocks were made by dehydrating in ethanol, rinsing in toluene, equilibrating in xylene and then, finally, embedding in paraffin. The intestine sections were then subjected to hematoxylin and eosin (H&E) staining. Villus length and the thickness of the muscle layer were measured using a light microscope (Nikon, Tokyo, Japan). 

### 4.4. Total Hemocyte Count (THC)

Hemocytes (500 μL per crab) were collected individually from the third pereiopod of crab, and then stirred and fixed with formalin (10%) for 10 min at room temperature. Then, the treated cells were stained using the Giemsa method and uniformly placed in a hemocytometer, forming a single layer without overlapping. Finally, the number of hemocytes was counted under an inverted microscope (Nikon, Tokyo, Japan). 

### 4.5. TUNEL Assay

TUNEL staining was performed to detect the apoptosis that occurred in the intestine of *E. sinensis* after FF exposure. Apoptosis cells were identified using the colorimetric TUNEL apoptosis assay kit (Beyotime, Shanghai, China). A Nikon Ti-E-A1R (Japan) fluorescent microscope was used to observe apoptosis. Cell nuclei were stained with DAPI and visualized in dark blue. Apoptotic cells were stained green. Six views of each sample were randomly picked, and total numbers of normal and apoptotic cells were counted respectively in view to calculate the apoptotic index. 

### 4.6. Antioxidant Enzyme Activities 

The intestines from control and from each FF exposure group were homogenized and centrifuged. Then, the concentrations of superoxide dismutase (SOD) and catalase (CAT) were measured with commercial assay kits (Jiancheng Institute, Nanjing, China). All results were normalized to total protein content in the respective sample for comparison. Each assay was run with five replicated samples.

### 4.7. Quantitative Real-Time PCR (qRT-PCR)

Total RNA was extracted from intestinal tissues using TRIzol reagent (Invitrogen, Waltham, MA, USA). The amount of RNA was measured by NanoDrop Spectrophotometer (Thermo Scientific, Waltham, MA, USA). Purified RNA was reversed into cDNA by HiScript^®^ Reverse Transcriptase (Vazyme Biotech, Nanjing, China) and then immediately stored at −20 °C for qRT-PCR. Expression of zonula occludens-1 (*ZO-1*), apoptosis- (*Caspase3*, *Caspase8*, *p53*, *Bcl-2* and *Bax*) and immune-related (*MyD88*, *ALF1*, *Relish* and *Dorsal*) genes was measured. Hieff^®^ qPCR SYBR^®^ Green (Yeasen Biotechnology, Shanghai, China) and a Roche LightCycler96 were used for qRT-PCR detection. *EF1-α* was used as an internal reference gene. Melting curves were generated to investigate the specificity of each amplified products. Relative gene expression was calculated by 2^−ΔΔCT^ method [54]. All samples were run in triplicates. Detailed primer sequences are presented in Table A1.

### 4.8. Intestinal Microbiome Analysis

#### 4.8.1. Total DNA Extraction and 16S rRNA Gene Sequencing

Total bacterial community DNA was extracted by using the Power Soil^®^ DNA Isolation kit (MoBio, Carlsbad, CA, USA) through the manufacturer’s instructions, and was then quantified for further use. Next, the bacterial full-length 16S rRNA gene was amplified with the primers 27-forward (5′-AGRGTTTGATYNTGGCTCAG-3′) and 1492-reverse (5′-TASGGHTACCTTGTTASGACTT-3′). Then, the purified PCR products were mixed in equidensity ratios and used to construct sequencing libraries by the PacBio sequencing platform (Biomarker-Technologies Company, Beijing, China). 

#### 4.8.2. Bioinformatics

SMRT-Link (V8.0) software was utilized to demultiplexed raw subreads and aligned it to CCS (Circular Consensus Sequencing) sequence. Sequences with more than 97% identity were clustered to one operational taxonomic unit (OTU) using USEARCH (V10.0). Chimeric and singleton sequences were also filtered. Data processing was conducted and displayed by R software (V3.5). Alpha diversity analysis, including richness abundance (Chao1 and ACE) and diversity (Simpson and Shannon) of communities, were implemented in QIIME (V2.0) software. Nonmetric multidimensional scaling (nMDS) method was performed based on Bray–Curtis similarity to evaluate the beta diversity in samples between groups. In addition, the relative microbial abundance and composition from phylum classification level to genus were also calculated. Significant differences of taxa in each group at phylum, family, and genus level were determined by metastats analysis. Eleven OTUs were chosen for heatmap comparison based on the significant abundance changes under FF-bath treatment on day 14 using the one-way ANOVA method.

#### 4.8.3. Microbial Function Prediction

Linear discriminant analysis (LDA) coupled with LDA effect size (LEfSe) was applied to find the biomarkers with the threshold of 4.0 [55]. PICRUSt was used to predict potential microbial metabolic function [56]. Finally, associations between biochemical indices and generic abundance were calculated by Pearson correlation analysis and shown in heatmap.

### 4.9. Statistical Analysis

All data were expressed as the mean ± standard errors of the means (SEM). Statistical analysis was carried out by one-way ANOVA, followed by LSD test, for the comparison between multiple groups using SPSS software (25.0, IBM, Chicago, IL, USA). Different letters (a, b, c, d) and asterisks “*” indicate significant difference among control and three florfenicol exposed groups (*p* < 0.05). Statistical results are shown in Supplementary Materials. 

## 5. Conclusions

In summary, FF exposure significantly affected *E. sinensis* gut health by damaging the intestinal barrier function, showing clear histological morphology variation and increased intestinal permeability. Meanwhile, exposure to FF induced and activated the antioxidant and apoptosis system to protecting cells from oxidative damage. Furthermore, the accumulation of FF in crabs resulted in declined microbial diversity and shifted community structure. Our results also suggest that the gut microbial diversity was closely related to crabs’ antioxidant and apoptosis systems, hence demonstrating their important roles in mitigating the effects of toxic stress on crabs’ intestine. Overall, this study highlights the essential role of intestinal homeostasis of crustaceans facing with antibiotic polluting systems.

## Figures and Tables

**Table 1:** Intestinal microbial diversity of that were exposed to FF for 14 days.

Groups	Chao1	ACE	Shannon	Simpson	Coverage (%)
D14_Control	71.00 ± 13.45	72.46 ± 9.07	3.78 ± 0.37 ^a^	0.87 ± 0.02 ^a^	99.91 ± 0.029
D14_LC	63.39 ± 12.25	66.03 ± 12.94	3.53 ± 0.17 ^ab^	0.82 ± 0.03 ^ab^	99.94 ± 0.018
D14_MC	70.33 ± 2.35	76.28 ± 2.67	3.58 ± 0.2 ^ab^	0.83 ± 0.02 ^ab^	99.91 ± 0.005
D14_HC	61.20 ± 13.94	63.08 ± 8.63	3.10 ± 0.37 ^b^	0.76 ± 0.06 ^b^	99.93 ± 0.013

Note: Values represent the mean Mean ± SE (n = 3). Values with the same letter within a line are not significant different (*p* > 0.05).

## Data Availability

All data are included in the manuscript and Supplementary Materials.

## Supplementary Materials

The following supporting information can be downloaded at: https://www.mdpi.com/article/10.3390/ijms24054412/s1.

## Appendix A

**Table A1:** Primer sequences for housekeeper gene and targeted genes.

Gene Name	Forward Primer (5′ to 3′)	Reverse Primer (5′ to 3′)
*Caspase 3*	AGTTTTGGGGAGGTGC	TCAGGTTCCGTGGGTT
*Caspase 8*	TGCTGGAGCTGGATGAATG	TTGGCCTCCACAGAGGTA
*p53*	AGCCATTCTCCTGTGGTAAAG	AGCCATTCTCCTGTGGTAAAG
*Bax*	GTCAGTGAACCTCAGCTGCAT	CACAGCCACATCACCCACGAA
*Bcl-2*	GCTCAGGGCAGCGTGT	GCAACCCAGACTCAATCAA
*Myd88*	GAACAGGATGCCATTGGTGAAA	GGTGATCTTGAGGTGGATGTAGAGT
*ALF1*	GCTGGCTGGACCGGATTATT	ATCACACGGGTGTTGCAGAT
*Relish*	TCTCCCTACTCTGACCATTCC	TTCCCACCATCTCACTCTTGT
*Dorsal*	CGTCAGCAGCACAGCAGAGAAT	CCCGTATTTCCTCCCTCAACTTCAG
*ZO-1*	AGTAGACCGGACCAGTAAG	GTGGGTTGACCACATCATAG
*EF-1α*	TTCCAGAAGTACGCTCCT	CAGCCTTGGTGGTCTTG
